# Monocular 3D Body Shape Reconstruction under Clothing

**DOI:** 10.3390/jimaging7120257

**Published:** 2021-11-30

**Authors:** Claudio Ferrari, Leonardo Casini, Stefano Berretti, Alberto Del Bimbo

**Affiliations:** 1 Department of Architecture and Engineering, University of Parma, Parco Area Delle Scienze 181/A, 43124 Parma, Italy; 2 Media Integration and Communication Center (MICC), Department of Information Engineering, University of Florence, Via di Santa Marta 3, 50139 Florence, Italy; leonardo.casini1@stud.unifi.it (L.C.); stefano.berretti@unifi.it (S.B.); alberto.delbimbo@unifi.it (A.D.B.)

**Keywords:** 3D body reconstruction, 3D modeling, learning 3D body shape parameters

## Abstract

Estimating the 3D shape of objects from monocular images is a well-established and challenging task in the computer vision field. Further challenges arise when highly deformable objects, such as human faces or bodies, are considered. In this work, we address the problem of estimating the 3D shape of a human body from single images. In particular, we provide a solution to the problem of estimating the shape of the body when the subject is wearing clothes. This is a highly challenging scenario as loose clothes might hide the underlying body shape to a large extent. To this aim, we make use of a parametric 3D body model, the SMPL, whose parameters describe the body pose and shape of the body. Our main intuition is that the shape parameters associated with an individual should not change whether the subject is wearing clothes or not. To improve the shape estimation under clothing, we train a deep convolutional network to regress the shape parameters from a single image of a person. To increase the robustness to clothing, we build our training dataset by associating the shape parameters of a “minimally clothed” person to other samples of the same person wearing looser clothes. Experimental validation shows that our approach can more accurately estimate body shape parameters with respect to state-of-the-art approaches, even in the case of loose clothes.

## 1. Introduction

Recovering the 3D structure of objects from monocular images is a long-standing problem in computer vision. In recent years, given the extraordinary development of new technologies, there has been an increasing interest in designing methods for recovering the 3D shape of humans. However, it becomes an extremely challenging problem if addressed without any assumption on either the scene or the objects themselves, i.e., in unconstrained conditions. Estimating the 3D shape of humans, either body, face or hands is further complicated by the highly deformable nature of this particular class of objects. To ease the problem, a popular workaround is to incorporate prior knowledge of the object to be reconstructed in the form of a statistical parametric model. One of the first examples of such a statistical model that has been used for human faces is the 3D Morphable Model (3DMM) [1]. Due to its solid theoretical formulation and interpretability of the results, this research topic is still actively researched with a recent renewed interest and several 3DMM variants proposed in the literature [2,3,4,5,6]. Given its potential, a similar methodology has been thereafter employed for developing other deformable models of, for example, hands [7], animals [8], and human bodies [9]. Given a deformable 3D human shape, either face or body, the possible applications are numerous, ranging from avatar animation [10,11,12] or garment generation [13,14], to pose estimation [15], view synthesis or data augmentation [16,17,18] or recognition of actions [19]. In all these cases, accurately recovering a faithful 3D shape is fundamental for optimal performance.

The particular task of reconstructing a 3D body shape from a single image involves solving two different sub-problems: recovering the (i) body shape and (ii) pose, i.e., spatial arrangement of some body joints. Despite being usually addressed together [20], the two can be also addressed separately, being one independent of the other [21].

One major difficulty in estimating a correct body shape arises because of clothing-induced occlusions, which likely hide the underlying body structure. The more loose the clothes are, the more difficult and ambiguous the retrieved body shape will be. This issue is particularly critical as it not only influences the shape estimation, but can also prevent a correct pose estimation. One possible way to account for this problem is that of generating photo-realistic 3D renderings; in this scenario, the ground-truth 3D shape is known, and can be used to learn how clothing affects the appearance, ultimately gaining invariance to it. However, this would either require collecting a large amount of data, or developing effective methods to augment a 3D body with variegated clothes, which is itself a challenging problem that is still being investigated [14,22].

In this work, we propose a simple yet effective solution to the task of recovering an accurate body shape under clothing. To this aim, we train a deep convolutional neural network to regress the shape parameters of a parametric model, the Skinned Multi Person Learning (SMPL) [9], from a single image. In particular, we design a particular training strategy: first, we train the network to regress the shape parameters using a synthetic dataset, where the ground-truth shape parameters are known. Then, to overcome the problem of limited real data with shape annotations, we estimate the shape parameters on real images of minimally clothed people using the trained network. We finally associate these parameters to other images of the same individuals with loose clothes to fine-tune the network in a self-supervised way. The intuition behind this solution is that the shape parameters of an individual should not change whether the person is wearing clothes, or not. This allows us to gain invariance to clothing, ultimately leading to a model that can effectively recover accurate body shape. Experimental results obtained on two datasets show a clear improvement in the accuracy of the reconstructed body shape with respect to existing solutions.

We instead do not account for the pose in our work, for two main reasons: first, if the two tasks are addressed jointly, as done in most of the literature, the level of accuracy that is obtained in each of the tasks likely diminishes. This because, even though pose and shape are treated independently in the model formulation, they do influence each other. Shape or pose ambiguities can result in wrong estimations that eventually will affect the other. Although obtaining an accurate model for human pose estimation on single images is made easier by the large availability of imagery annotated with body joints, the same does not hold for the 3D body shape. For these reasons, we focus on solving the latter problem.

In summary, the main contributions of our work are:

- We propose a simple yet effective solution for reconstructing the body shape under clothing from a single image. The main novelty is in the proposed idea of imposing the constancy of the parameters that deform the template model to the target body image with both *minimal* or *normal* clothing;
- We also demonstrate how synthetic data can be leveraged to build a model with a sufficiently reliable estimate for minimally clothed bodies, and that we can use the estimated parameters to automatically label other images of the same person wearing clothes, ultimately improving the shape estimation.

The rest of the manuscript is organized as follows: In Section 2, we summarize the work in the literature that are closer to our proposed solution; In Section 3, we introduce the SMPL body shape model; Using SMPL, we present our approach for estimating the body shape under clothing in Section 4; In Section 5, we provide an experimental validation of our method on two datasets that clearly shows its effectiveness when compared to the methods in the literature for the task of body shape estimation under clothing; Finally, conclusions and possible future work directions are drawn in Section 6.

## 2. Related Work

The task of reconstructing the body shape from single or multiple images can involve two different sub-tasks: the estimation of the 3D body shape, and the pose, expressed in terms of joint locations. Most of the works in the literature address the two problems jointly; even though we are only interested in recovering an accurate shape independently from clothing or pose, in the following we provide a comprehensive review of the recent works in the literature, which can be divided in two main categories: *model-free* and *model-based*.

Model-free approaches try to directly infer a 3D structure from the image, and generally use different representations for 3D data. Among the few methods in this category, we mention BodyNet [23] and DecoMR [24]. BodyNet [23] uses a Voxel-CNN to estimate a volumetric representation of the body, thus resulting somewhat constrained to the intrinsic limitations brought by voxel-based representations. DecoMR [24] first recovers an UV map from a single image, then uses an additional module, called locationNet, to produce a location map that is generated from the UV map and a reference mesh. The SMPLify++ [25] approach, instead, leverages the semantic segmentation task to detect a semi-dense set of landmarks that are used to retrieve a coarse shape and the pose. All these methods do not rely on a parametric model, and so have the potential advantage of being not bounded by the expressiveness of the model. On the other hand, a drawback of such solutions is that the resulting reconstruction needs post-processing operations for further manipulation, and the resulting shape might be inconsistent or noisy.

Model-based methods make use of some parametric model as a statistical shape prior. In the literature, some different shape models have been proposed, starting from the SMPL [9], to GHUM [26], which extends the SMPL with face and hand models, or the STAR [27], which is capable of modeling local body shape deformations. Many methods develop on the top of such models. One of the first examples is the HMR [20] method, which uses a deep encoder to regress pose, shape and camera parameters of a SMPL body model from a single image, optimizing with respect to ground-truth body joint locations. Its simple formulation is effective, even though sub-optimal performance is obtained for each separate task. DensePose [15] also uses the SMPL model at training time to estimate a dense pose of bodies in the wild. Despite recovering accurate dense poses, the method is not really suitable for estimating the body shape. The opposite problem is solved by the Tex2Shape [28] method that uses a pix2pix-like network to infer normal UV displacement from a single image. This is then put on the top of an SMPL body model to reproduce clothing details. By contrast, the SPIN [29] method uses the SMPL model but optimizes with respect to the mesh instead of joint locations, which allows for a more accurate shape recovery. The lack of real images with the corresponding ground-truth 3D mesh makes it complex to effectively train this architecture.

With respect to the above-mentioned works, our solution focuses on the task of recovering accurate SMPL shape parameters from single images of people that could wear loose clothes. We use a similar architecture to that of HMR, but focus on how to solve the problem of missing ground-truth data for real people, without using joint locations. Other similar methods that aim at the same goal are the “Naked Truth” method of Bualan et al. [21], which uses multiple poses to infer shape consistency under clothing under the assumption that shape parameters are independent from pose parameters. A drawback of this approach is that it requires multiple images to solve the task. The other related methods are the one of Wuhrer et al. [30], and Hu et al. [31]. Both, however, estimate the shape under clothing directly from single or multiple 3D scans instead of images.

## 3. SMPL

Our proposed method falls into the category of model-based solutions for 3D body shape reconstruction. For such approaches, the identification of a suitable 3D body template model is important also in relation to the particular task. We identified the SMPL as the most appropriate 3D body shape model for our purposes. For the sake of completeness and for a reference for the presentation of our approach, the main features of SMPL are summarized below.

**Figure 1:**
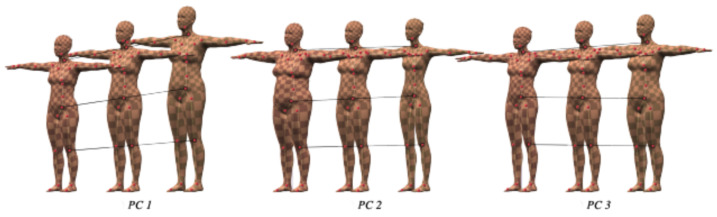
Example of the first 3 principal components of shape variations for the SMPL model.

Skinned Multi Person Learning (SMPL) is a skinned vertex-based model that accurately represents a wide variety of body shapes in natural human poses. The parameters of the model are learned from data including the rest pose template, blend weights, pose-dependent blend shapes, identity-dependent blend shapes, and a regressor from vertices to joint locations. The SMPL is a statistical model that encodes the human subjects with two types of parameters:

- Pose parameters $\alpha_{i}$: a pose vector of 24×3 scalar values that keeps the relative rotations of joints with respective to their parameters. Each rotation is encoded as an arbitrary 3D vector in an axis-angle rotation representation;
- Shape parameters $\beta_{i}$: a shape vector of 10 scalar values, each of which could be interpreted as the amount of expansion/shrink of a human subject along some direction such as taller or shorter. The shape variations are learned by applying PCA on the set of training bodies. Some examples are shown in Figure 1.

SMPL is a differentiable function that returns a triangular mesh with N=6980 vertices, $M\left(\theta ,\beta \right)\in R^{3\times N}$, which is obtained by modeling the vertices of the template body based on β and θ, then articulating the bones according to the rotation of the joints θ, and finally deforming the surface with a linear blend skinning. Given its potential to represent many human meshes with only 82 parameters, this model is widely used in model-based methods.

In this work, particular attention has been paid to the estimation of the shape parameters from single images, neglecting the body pose. We are in fact interested in reconstructing a faithful body shape in the particular case where people might wear loose clothes. Even though this could also influence the pose estimation, we focus on the former problem. Despite the two sets of parameters being usually estimated jointly, the two are independent. We argue that accurately estimating the shape parameters regardless changes in pose or clothing, can also help in subsequently estimating a more accurate location of the joints.

## 4. Proposed Method

**Figure 2:**
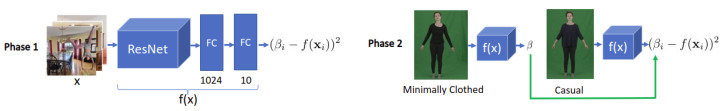
**Phase 1**: the proposed model takes as input an image of a person and regresses the SMPL shape parameters β. **Phase 2**: the trained model is used to extract β parameters from the minimally clothed images. The parameters are assigned to each image of the same individual and the network if fine-tuned.

Our proposed method is based on the SMPL model, but can potentially be applied with any parametric body model. Our goal is to estimate SMPL shape parameters from a single RGB image *I* containing a person. To this aim, we consider a ResNet-50 [32] architecture pre-trained on ImageNet [33]. As with the HMR method [20], we stack a new 1024-dimensional fully connected layer and an additional 10-dimensional layer to regress the SMPL shape parameters on top of the architecture. The two fully connected layers are trained from scratch, while the rest is fine-tuned. A sketch of the architecture is shown in Figure 2, left.

In this work, we are particularly interested in recovering a faithful shape of the body. Given that shape and pose do not depend on each other [21], here we neglect the pose and train the network using a simple mean square error (MSE) loss on the shape parameters. Formally, the network can be regarded as a function $f\left(x\right):X\to R^{10}$ that maps an input image x∈X to a set of shape parameters $\beta \in R^{10}$. Using this notation, we train the network towards minimizing the following:

(1) $$L=\frac{1}{N}\sum_{i=1}^{N}{\left(\beta_{i}-f\left(x_{i}\right)\right)}^{2}\;,$$

where *N* is the mini-batch size, and $\beta_{i}$ are the ground-truth parameters corresponding to the image $x_{i}$. The main problem with this strategy is the lack of data with associated ground-truth parameters. To obtain faithful ground-truth parameters, the images necessarily need to be either synthetically rendered using a SMPL-generated body, or associated with a 3D mesh so that the parameters are obtained by fitting the SMPL to the mesh. In the former case, it is well known that learning only on synthetic data can lead to poor performance on real data, whereas using mixed data can be beneficial [34]. On the other hand, collecting a sufficient number of real images associated with 3D scans is equally complex because of the burdensome process of 3D scanning. Furthermore, 3D scanners will also capture the clothing, making such not suitable for our purposes.

### 4.1. Training Process

Our major contribution is related to how to address the above problem. The idea is that of training the network in two separate phases. First, we use synthetic data generated by rendering a SMPL body with clothing onto real natural images to perform an initial training. To this aim, we used the SURREAL dataset, which contains video sequences of 3D rendered people on real backgrounds. This allows us to be somewhat robust to different clothing since the ground-truth parameters are known. We refer to the latter process as “Phase 1”. However, since clothing is also rendered, it results tight to the body and does not hide the silhouette, as instead happens with real clothing. We empirically learned that using synthetic data is not enough to obtain sufficiently accurate results on real images.

To increase the generalization performance to real images that instead are not labeled with such parameters, we propose to use real images of people with different clothing. In particular, we first obtain the shape parameters from a “minimally clothed” version of each subject. Then, following a self-supervised scheme, we associate the estimated parameters to any other image of the same individual. This idea grounds on the assumption that the shape parameters should not change even in case the person is wearing loose clothes, given that the parameters encode the body shape. If the model is sufficiently accurate on the minimally clothed images, then we can fine-tune the model on real clothed people ensuring the shape parameters will be consistent, ultimately gaining a certain degree of invariance to clothing. We refer to this as “Phase 2”. The whole process is shown in Figure 2.

## 5. Experimental Results

In this section, we first introduce the datasets used in the experiments for training and test, and provide training details of the proposed architecture. Then, we report the results of our experimental validation.

### 5.1. Datasets

In our experiments, we used two datasets: SURREAL and People Snapshot, The main characteristics of the datasets are detailed below.

**SURREAL:** The Synthetic Humans for Real Task dataset is a large-scale person dataset containing around 6M video frames of synthetic humans. The images are photo-realistic renderings of people under large variations in shape, texture, viewpoint, pose and backgrounds. It comprises 145 subjects, and a pre-defined train/test splitting: the training set consists of 115 subjects, for a total of 5M frames. The test set is instead composed of 30 subjects and 1M frames. The synthetic bodies have been created using the SMPL body model, and so the ground-truth parameters are known. This dataset is used in Phase 1 of our method to train the model towards regressing the SMPL shape parameters β.
**People Snapshot:** The People Snapshot dataset consists of 21 sequences of 12 subjects, 6 males and 6 females, varying a lot in height and weight. The sequences are captured with a fixed camera where subjects rotate themselves while holding an A-pose. To cover a variety of clothing, lighting conditions and background, the subjects were captured with varying sets of garments and with three different background scenes: in the studio with green screen, outdoor, and indoor with complex dynamic background. Each of the 12 subjects is recorded with one or more clothing. We use this dataset for the second phase of our method. Since there is no pre-defined splitting in this dataset, we used 10 subjects for training and 2 for testing in a cross-validation setting, for a total of around 5 K frames for training and 500 for testing.

### 5.2. Training Details

The training has been carried out using a single NVIDIA TitanX GPU. To train the Phase 1 on the SURREAL dataset, considering the high redundancy of the frames, we subsampled the training set using 1 frame every 3, for a total of around 350 K images. Simple random horizontal flipping and translation were used for data augmentation. We used a batch size of 64 and trained the model for 100 epochs.

**Figure 3:**
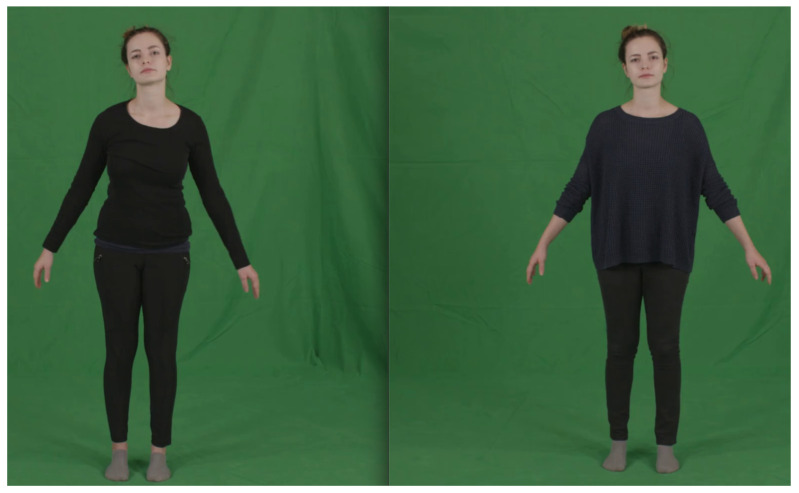
Samples of a same subject in the People Snapshot dataset. The “minimally clothed” version (with tight clothes) is shown on the **left**, and is used to estimate the shape parameters. These parameters are then assigned to the other sequences with looser clothes (**right**).

For the Phase 2, we fine-tuned the model on the training splits of the People Snapshot dataset for 50 epochs. To obtain the ground-truth annotations as described in Section 4.1 we selected, for each subject, the sequence that corresponds to the minimally clothed version, which is associated with the “sport” clothing class. We use the model resulting from Phase 1 to estimate the SMPL shape parameters on these sequences. Finally, these parameters are associated with all the remaining sequences and used to train the model using the loss function in Equation (1). An example of the difference between the garments in this dataset is shown in Figure 3.

### 5.3. Results

Here we report quantitative evaluation of our method compared to recent state-of-the-art approaches. Most methods report results in terms of Mean Per Joint Error (MPJE); however, we do not account for the pose and are interested in recovering an accurate 3D body shape. Therefore, we evaluate in terms of Mean Per Vertex Error (MPVE), which is computed as the average Euclidean distance between the deformed SMPL model and the ground-truth. As per the standard convention, the models are first put in T-pose.

First, we report in Table 1 results obtained on the test set of the SURREAL dataset, comparing against both model-free and model-based approaches. Our method obtains a significant improvement over other works. This is somewhat expected, since we do not account for the pose, while the compared methods do. However, this experiment served us to verify that we can obtain sufficiently accurate body shape parameters to be used for the second phase, and represents a piece of evidence that it can be beneficial to address the two problems separately.

In Table 2, we report results on some test subjects of the People Snapshot dataset, comparing the reconstruction accuracy obtained by our method before and after fine-tuning. Results are compared with those obtained by the recent HMR [20] approach, which is the most similar to our proposed solution. Results show that after the fine-tuning process, our method achieves more accurate reconstructions. In particular, Table 2 (bottom) highlights that our training strategy narrows the gap between the reconstruction errors obtained from images of people in different clothing. These results suggest us that the proposed solution of re-using the shape parameters estimated from a minimally clothed version of the subject is effective, and represents a viable solution to address the difficulty of obtaining sufficient amounts of annotated data for training a body reconstruction model. However, to do so, a model that can accurately recover the shape at least in case of tight clothes is of fundamental importance for reliable results.

**Figure 4:**
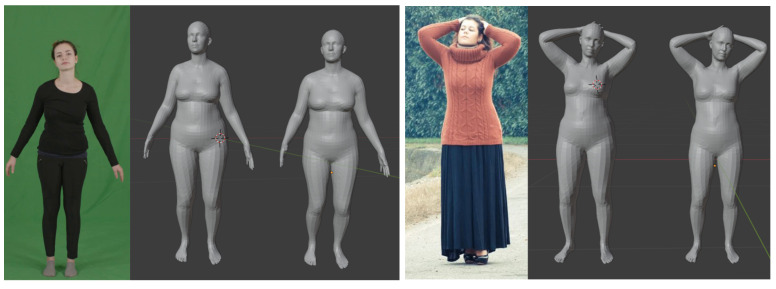
Qualitative examples of body reconstruction from an image of the People Snapshot (**left**) and an “in the wild” image (**right**). The two reconstructions are obtained using the HMR method (**left**) and our approach after Phase 2 (**right**).

**Figure 5:**
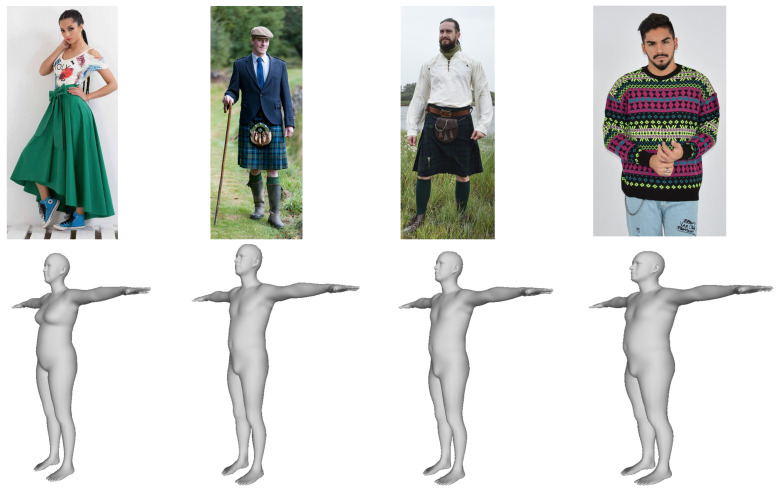
Qualitative reconstruction examples from “in the wild” images collected from Internet.

In Figure 4, we report two qualitative reconstruction comparisons, one from the People Snapshot dataset, and the other obtained from an “in the wild” image collected from Internet. The pose parameters to render the mesh were obtained with HRM. Even though we cannot compute quantitative measures, we can observe that the results obtained with our method better reflect the body shape. In particular, some unrealistic details, such as the abrupt change in the pelvis shape resulting from the HMR method, are corrected with our model. Clothes induced occlusion not only prevent a correct estimation of the body shape but can also change the silhouette shape significantly. Methods that rely on the visual data to fit the model would suffer from this, as shown in Figure 4. Our model, instead, even though it might not be always able to recover a perfect shape when it is completely occluded, is less affected by this problem. Ultimately, this is also beneficial to recover the pose more accurately as they normally depend on each other. Finally, in Figure 5, we report some other qualitative reconstruction examples (in T-pose) obtained with our method from images collected randomly from Internet. Despite the unusual and challenging clothes worn, incomplete bodies (rightmost example) and variegated poses, the reconstructions are stable, and provide a reasonably accurate estimation of the shape even for unseen samples such as men wearing a kilt.

### 5.4. Discussion

Results reported so far highlight that synthetic data can be leveraged to train a model that can estimate sufficiently accurate shape parameters from real images of people wearing tight clothes. This allowed us to define an incremental training strategy that relies only on the model itself to label new data, and increase the estimation accuracy on more challenging cases. We reported results on the People Snapshot dataset since each body image is associated with a ground-truth 3D model, allowing us to quantitatively evaluate the improvement obtained. Nonetheless, given its proved efficacy, this strategy can be further extended to potentially any other dataset, bringing the advantage of significantly simplifying the data collection process. This represents an efficient yet effective way to collect labeled data to train more accurate models in a self-supervised way. Other way around, our proposal suggests that one could collect real data without the need for a 3D scan for each sample, overcoming the burden of 3D data collection and processing. Instead, one can acquire a 3D scan of the minimally clothed person, and then collect a large amount of other clothed images and associate the same shape parameters to all of them.

## 6. Conclusions and Future Work

In this work, we presented a strategy for training a deep CNN towards estimating the shape parameters of an SMPL body model from single images, which is robust to clothing occlusions. Our solution grounds on the idea that the shape parameters of an individual should not change if loose clothes are worn. We propose to first train the network on synthetic data so to learn the mapping from image to shape parameters, neglecting the pose. In this way, the network can recover sufficiently accurate shape parameters. We then propose to use this network for estimating parameters from real images of minimally clothed people, and associate these parameters to other images of the same subjects with clothes. This allows us to fine-tune the network on real data, ensuring the target parameters are accurate enough to meaningfully train the network.

Experimental results showed a promising direction towards developing robust shape estimation models. In this work, to quantitatively evaluate the performance of the model, a small dataset with real people with associated ground-truth 3D scans was employed. However, the positive outcomes of our validation suggest that broadening the same strategy to larger, non-annotated datasets is feasible, which would likely lead to even more robust models. In addition, to fairly validate our strategy against previous methods, we restricted our investigation using a simple MSE loss to regress the parameters. We believe more complex training strategies and architectures could further push the performance of this simple model.

## Figures and Tables

**Table 1:** Quantitative results on the SURREAL dataset in terms of MPVE (mm).

Type	Method	Error (mm)
Model-free	SMPLify++ [25]	75.31
	Tung et al [35]	74.52
	BodyNet [23]	73.63
	DecoMR [24]	56.37
Model-based	Neural Body Fitting [36]	65.8
	SMPLR [37]	62.3
Model-based	Our approach	25.43

**Table 2:** Quantitative results on some test samples of the People Snapshot dataset in terms of MPVE (mm). Table in the “All garments” side reports average reconstruction errors compared to HMR (averaged over all garments). Our method after fine-tuning obtains accurate reconstructions. Table in the “Tight-Loose” side highlights the improvement obtained on loose clothes (casual) after fine-tuning the model with parameters associated with the minimally clothed version (sport).

Method	Subject	Error (mm)
	All garments	
Ours—Phase 1	Female 1	22.77
Ours—Phase 2	Female 1	17.77
HMR [20]	Female 1	22.23
Ours—Phase 1	Female 3	25.93
Ours—Phase 2	Female 3	10.74
HMR [20]	Female 3	11.37
Ours—Phase 1	Female 6	30.63
Ours—Phase 2	Female 6	20.23
HMR [20]	Female 6	23.87
Ours—Phase 1	Male 9	46.75
Ours—Phase 2	Male 9	42.55
HMR [20]	Male 9	42.57
	Tight-Loose	
Ours—Phase 1	Female 1—Sport	22.27
Ours—Phase 2	Female 1—Sport	17.69
Ours—Phase 1	Female 1—Casual	25.38
Ours—Phase 2	Female 1—Casual	17.83

## Data Availability

Datasets used in this research are publicly available as indicated in the related sections.
